# Online health information seeking behaviours of parents of children undergoing surgery in a pediatric hospital in Rome, Italy: a survey

**DOI:** 10.1186/s13052-020-00884-7

**Published:** 2020-09-29

**Authors:** Luisa Russo, Ilaria Campagna, Beatrice Ferretti, Elisabetta Pandolfi, Marta Luisa Ciofi Degli Atti, Simone Piga, Sally Jackson, Caterina Rizzo, Francesco Gesualdo, Alberto E. Tozzi

**Affiliations:** 1 Multifactorial and Complex Diseases Research Area, Bambino Gesù Children’s Hospital, Piazza di Sant’Onofrio 4, 00165 Rome, Italy; 2 Clinical Epidemiology Unit, Bambino Gesù Children’s Hospital, Piazza di Sant’Onofrio 4, 00165 Rome, Italy

**Keywords:** Children, Surgery, Internet, Information search

## Abstract

**Background:**

People increasingly search online for health information. Particularly, parents of patients often use the Internet as a source for health information. We conducted a survey to investigate the online searching behavior of parents of patients < 18 years, admitted for surgery in an Italian pediatric hospital.

**Methods:**

The cross-sectional survey was nested in a prospective cohort study on surgical procedures. Parents of patients undergoing surgical procedures at Bambino Gesù Children’s Hospital, Rome, Italy, were enrolled and contacted by phone after the procedure. We recorded socio-demographic data, sex, length of stay following surgery, proximity of residence to the hospital, use of the internet to search for information on the surgery before and after the intervention and effect of information found online.

**Results:**

The majority (91%) of parents of children undergoing surgical intervention used the internet. Of these, 74.3% of parents searched for information before surgery, and 26.1% searched for information after. Most parents searched for information on the care provider’s website. Two thirds of parents reported that information found online had increased their understanding of the child’s condition. Multivariate analyses indicated that families living far from the hospital (> 43 km) were more likely to search for health information (OR 2.3; 95% CI 1.34–4.00), as were families of patients undergoing a major surgery (OR = 2.1; 95% CI 1.04–4.11).

**Conclusions:**

Parents of children undergoing surgery often search online for information on their child’s intervention, in particular those whose child is scheduled for a major surgery and those living far from the hospital. A survey like the present one allows to understand parents’ information needs, to better guide them in online information seeking and to better tailor information provided on the care provider’s website.

## Background

In 2019, 92% of EU-28 households had access to the internet [1], including 88% of households in Italy [2]. Widespread use of the internet has transformed the way we access health information. There is clear evidence that people increasingly search online for information on a wide range of clinical topics [3, 4].

Research suggests that parents seeking information about their child’s health online is a consolidated behavior [5–8], with the most recent studies in Western Europe reporting that 80–90% of parents use the internet in this way [9–11]. Parents report seeking online information about specific conditions and symptoms their child is experiencing [12, 13], medical and surgical treatments [10, 14, 15], and for more general parenting advice [12]. Information found online can influence questions parents ask their child’s clinicians and decisions they make about their child’s care [7, 10, 16].

Previous studies of parents’ health information seeking patterns have concluded that several factors influence searching behaviours, including positive/negative perceptions of online information and social/cultural contexts [17, 18]. Parents who have a higher level of education [10, 11], are female [11], have younger children and/or children with acute diseases [19], private health insurance, daily internet use, and access to a smartphone [10] have been found to seek information online more often.

Parents regard online health information as easier to access and more up to date than offline resources and often trust the information they find [16, 20], although some studies have raised questions about the quality of online health information [21–23].

A number of studies have investigated online information seeking by parents of children undergoing surgery [10, 14–16, 24–26]. These studies focussed on various aspects of parental health information seeking, including sources of online information [15, 16, 24], what parents search for [14, 16], perceptions of quality/usefulness of information [14–16, 24, 25], comparisons with traditional sources of information (e.g. surgeons) [10, 14, 15], how parents use information they find (e.g. to aid decision making, discuss with clinicians), and determinants of searching behaviour (e.g. parent gender, education level) [10]. All the studies found that parents frequently access information about their child’s health online and/or had accessed information about their child’s specific surgery before the procedure took place.

The current study aims to build on this body of knowledge by assessing the online health information seeking behaviours of parents of patients undergoing elective and emergency surgeries at a large paediatric hospital in Italy. In addition, the study explored the role of the hospital’s website in online information seeking and how this could be improved to best meet parents’ information needs.

## Methods

### Study design, setting and patient population

This cross-sectional analysis forms part of a wider prospective cohort study investigating surgical site infections in children, which is described in further detail elsewhere [27]. The cohort included children aged 0–17 years undergoing surgical procedures without implants at Bambino Gesù Children’s Hospital (OPBG), a 4-site 600-bed paediatric hospital in Rome, Italy. Patients were enrolled consecutively from surgical procedure lists (elective and emergency) for one index week in March 2018.

### Data collection

Patient data were extracted from clinical records, including age, gender, complexity of surgical intervention (major/minor, measured using Agency for Healthcare Research classifications [28]), length of stay following surgery, and proximity of patient’s residence to the hospital.

Data relating to internet use were collected 30 days post-discharge via telephone interview with patients’ parents. Questions on internet use were included in a wider survey about surgical site infections, which was administered by trained nurses. Questions focused on whether parents searched the internet for information about their child’s surgery before/after the procedure and, if they did, how information they found affected them (improved their understanding of their child’s problem, increased their anxiety over their child’s problem, and/or improved the management of their child’s problem). A subset of questions focused specifically on the role of the care provider’s website (http://www.ospedalebambinogesu.it) in meeting parents’ information needs and how this could be improved. These concepts are summarised below:

- Searching for information on the procedure *before* surgery
  - General internet searches
  - Searches on care provider’s website
- Searching for information on the procedure *after* surgery
  - General internet searches
  - Searches on care provider’s website
- Perceived effects of information found through internet searches (both general and on care provider’s website)
- Ways in which care provider’s website could better meet the information needs of parents

Survey questions were piloted in a small sample of parents to assess readability and question construction. Survey data were anonymized and recorded in a secure database (Microsoft Access 2007–13).

### Data analysis

Survey data were collated and analysed using descriptive statistics. Proportions were calculated using as denominator either the total respondents or the participants that reported using the internet for information on surgery, as appropriate. Relationships between potential predictors (patient age, gender, complexity of surgical intervention, length of stay, and proximity to the hospital) and parents’ internet searching behaviours (i.e. whether or not they searched for information on the internet) were analysed statistically. Fisher exact and χ2 tests were used for categorical variables, and t-tests and Mann-Whitney tests for continuous variables. Multivariate logistic regression was performed, adjusting for potential confounders identified through univariate analyses, to determine whether patient characteristics (age, complexity of procedure, and distance of residence from hospital) were associated with parents’ searching behaviours before their child’s surgery. For continuous variables (age and distance of residence from the hospital), median values were used to split the sample into two groups (e.g. aged < 7 years and ≥ 7 years) in order to calculate the relative odds of searching for information before the surgery. Statistical analyses were carried out using STATA, Statistical Software: Release 13. College Station, Tx: StataCorp 2013.

We also analyzed how distance between patients’ residence and hospital site affected online searching behavior. Proximity of patients’ residences to the hospital site was analysed using Open Route Service (ORS) and validated through other online mapping services.

## Results

In the index week of the study, there were 306 consecutive surgical procedures performed in the hospital on patients aged 0–17 years. Among these patients, 4 had an address outside of Italy and 57 families could not be contacted by phone after 30 days, resulting in a sample of 245 patients whose families participated in telephone interviews. None refused to participate.

Table 1 shows a summary of patient characteristics, stratified by parents who reported using the internet and parents who reported not using the internet. The majority (91%, *n* = 222) of parents of children undergoing surgical intervention at OPBG reported using the internet. No differences were observed between the groups in terms of child’s age, gender, complexity of procedure, whether they lived outside the municipality of Rome, and distance of their residence from the hospital.

**Table 1:** Demographic and clinical characteristics of patients by parents’ internet searching behaviors

Characteristic	Parents did not report using the internet	Parents did report using the internet	***p***-value
Number of all patients’ parents interviewed, n (%)	23 (9.4)	222 (90.6)	< 0.001
Age of the patients in years, median (IQR)	6.2 (2.0–9.7)	7.0 (3.6–11.3)	0.380
Patient age groups, n (%):			0.830
0–4 years	9 (39.1)	83 (37.4)	
5–10 years	10 (43.5)	81 (36.5)	
10–14 years	2 (8.7)	36 (16.2)	
15–18 years	2 (8.7)	22 (9.9)	
Patient’s gender, n (%)			0.703
Male	14 (60.9)	144 (64.9)	
Female	9 (29.1)	78 (35.1)	
Length of hospital stay in days, median (IQR)	4 (1.0–19.0)	4 (1.0–17.0)	0.345
Complexity of surgery based on AQHR, n (%)			0.856
Major	18 (78.3)	170 (76.6)	
Minor	5 (21.7)	52 (23.4)	
Living outside the municipality of Rome, n (%)	7 (30.4)	90 (40.5)	0.345
Distance from the hospital in Km, median (IQR)	62 (32–248)	43 (24–148)	0.177

Table 2 summarises the searching behaviours, the effect of the information and the expectations for the hospital’s website among parents responding to the survey who reported that they did use the internet (*n* = 222). Almost 75% reported searching for information about their child’s surgery on the internet *before* the procedure and 65% reported searching on the care provider’s website. Twenty-six percent of patients’ parents reported searching for information on the internet after discharge and 21.6% visited the care provider’s website. Two thirds (66%) of parents performing searches on the internet relative to the surgery reported that information found on the internet had improved their understanding of their child’s condition, while almost one third (30.9%) felt that it had improved the management of their child’s condition. A low proportion of parents (8.5%) reported experiencing increased anxiety as a result of information found on the internet.

**Table 2:** Internet searching behaviours reported by parents and effects of information found

Survey question	Number of parents answering ‘Yes’ (%)
Did you search for information on the internet about your child’s surgery *before* arriving at the hospital? ^a^	165 (74.3)
Did you visit the care provider website *before* arriving at the hospital? ^a^	145 (65.3)
Did you search for information on the internet about your child’s surgery *after* discharge? ^a^	58 (26.1)
Did you visit the care provider website *after* discharge? ^a^	48 (21.6)
What has been the effect of the information you found on the internet (multiple responses allowed)? ^b^	
It has improved my understanding of my child’s condition	109 (66)
It has improved the management of my child’s condition	51 (30.9)
It has increased my anxiety over my child’s condition	14 (8.5)
Other	3 (1.8)
What information would you like to find on the care provider website? (multiple response allowed) ^a^	
Contact information for clinicians	65 (29.3)
Copies of medical records	65 (29.3)
Information on hospital access	46 (20.7)
Information on patient pathways	44 (19.8)
Information on your child’s surgical intervention	25 (11.3)
Information on warning signs and complications associated with the surgical intervention	25 (11.3)
Other	8 (3.6)

Denominators in the proportions vary by question:

^a^: all parents who used the internet, *N* = 222

^b^: all parents who searched online for information regarding surgery, *N* = 165

Parents reported a range of information needs from the care provider’s website. The most common types of information parents reported that they would like to find were contact details for clinicians (29.3%) and copies of medical records (29.3%). Parents were also interested in information on hospital access and patient pathways (20.7 and 19.8%, respectively) and, to a lesser extent, information on surgical interventions (11.3%) and associated complications (11.3%).

The results of the multivariate logistic regression analysis, shown in Table 3, indicate that parents of patients living farther from the hospital (≥44 km) were twice as likely to search online for information about their child’s surgery before the procedure than those who lived closer to the hospital (OR = 2.3 [95% CI = 1.34–4.00], *p* = 0.003). It also appeared that parents of patients undergoing a more complex surgery may be more likely to search for information online than those undergoing a minor surgery (OR = 2.1 [95% CI = 1.04–4.11], *p* = 0.037).

**Table 3:** Results of multivariate logistic regression analysis of patient characteristics associated with searching for information on the internet *before* the surgery

Variable	Adjusted OR (95% CI)	***p***-value
Patient’s age		
< 7 years	1.00	
≥ 7 years	1.43 (0.81–2.53)	0.222
Complexity of procedure (based on AQHR)		
Minor	1.00	
Major	2.07 (1.04–4.11)	0.037
Presence of chronic disease		
No	1.00	
Yes	0.83 (0.43–1.59)	0.571
Distance of residence from hospital		
< 44 km	1.00	
≥ 44 km	2.31 (1.34–4.00)	0.003

*AQHR* Agency for Healthcare Research classification, *CI* confidence interval, *km* kilometers, *OR* odds ratio

## Discussion

This study emphasises the importance of the internet in the information seeking behaviours of parents whose children are undergoing surgery. Over a half of parents who participated in the telephone survey reported searching online for specific information about their child’s surgery *before* they were admitted, while around a quarter reported performing these searches *after* the surgery had taken place.

Previous studies into the information seeking behaviours of parents whose children are undergoing surgery have found that around 40–98% of parents search online for information about their child’s condition [10, 14–16, 24–26]. This study is the first to stratify parents depending on whether they searched for information online *before* or *after* their child’s surgery. The finding that parents were more likely to seek information online *before* surgery makes sense given that parents are likely to want to know more about their child’s condition, what the procedure will involve, and potential complications before surgical treatment is administered.

Data on pre-surgery information needs obtained through a survey like the one presented in this article could serve as a basis to tailor the kind of information provided to parents before surgery, which can be delivered through different means: during face-to-face encounters with clinicians; through paper leaflet provided to parents including a tailored description of the intervention; through explanations of surgical procedures using novel technologies, like 3D printing or virtual reality [29]; directing parents to reliable web-based information, including a specific indication to access the hospital’s website.

Previous studies have found that 17–18% of parents report that information found online has influenced their decisions about their child’s healthcare [7, 10]. Public search engines (e.g. Google) are often the starting point for parents looking for information about their child’s health [9, 14, 16, 19, 24]. However, search engines do not screen results on the basis of directing parents towards reliable medical information, and concerns have been raised over the quality of online health information accessed by parents [10, 14, 21–23]. Care provider websites, on which contents are written by clinicians, are used less frequently [11] but are considered by parents to be an accurate, reliable source of information [5, 24]. It has been suggested that the best way to ensure parents have access to high-quality information about their child’s health is to meet their information needs via care provider websites, where clinicians can ensure that relevant, accurate, and up to date information is displayed [10, 14]. The majority of parents had visited the care provider’s website before their child’s surgery, substantially more than in previous studies where less than 20% of respondents used their care provider’s website [5, 11, 24]. This higher proportion compared to that reported in previous studies could be partially explained by the high reputation of the OPBG website at the national level. Search engine optimization of the informative pages of the website, allowing specific pages to get a higher rank on the search engine result page, could have increased access to the hospital website for information on the procedure. Moreover, we collected a detailed description of the reasons why parents accessed the hospital’s webpage, and we found that a large proportion of them were interested in finding clinicians’ contact information and copies of medical records online. These insights will inform a redesign of the hospital website, to help parents find the information they need more easily.

A more detailed profiling of parents that were more likely to search for information online can facilitate an improved targeting of the information provided on the hospital websites or on reliable websites where parents can be directed to. Those that were more likely to search for online information were parents of children undergoing a more complex surgery, possibly because parents’ perceived risks of the surgery were greater; and parents of children who lived farther away from the hospital, possibly because they had less chances of having face-to-face contacts with the clinicians. Neither of these determinants of parental internet searching behaviours (more complex surgeries and living farther from the care provider) has been reported previously.

More than 60% of parents in this study felt that the information they found online had improved their understanding of their child’s condition requiring surgery, compared to 84–92% of parents in previous studies [14, 15]. This prompts the need to improve accessibility and quality of information on surgery dedicated to parents. A small proportion of parents said that online information had increased their anxiety over their child’s condition. This may relate to findings, reported in previous studies, that some parents found online information to be ‘distressing’ [14]. This result reinforces the need of guidance by and feedback with clinicians or with family pediatricians regarding information found online. Around 30% of parents felt that information found online had improved the management of their child’s condition, perhaps because they discussed this information during consultations with clinicians, or because online information provided practical, easy-to-use guidance to manage the child before or after surgery.

This study has a number of limitations. First, we did not collect data about parents who did not respond to the survey, and this may imply a selection bias. Moreover, as with any survey where some respondents did not answer all of the questions, there was potential for self-selection bias in the sample of parents answering some questions. Data on online health information seeking refers to the parents of patients rather than the patients themselves. Children and adolescents also seek health information online [30] and it would be worth considering them in future research, particularly because there is evidence to suggest their information-seeking behaviours differ from those of their parents [25]. Finally, data were not collected on the socio-demographic characteristics of parents (e.g. gender, education level), as has been the case in some previous studies.

## Conclusions

In conclusion, the present study describes the online searching behaviour of a specific population, i.e. parents of children undergoing surgery. Parents often use the internet as a source of information on their child’s intervention, in particular before surgery. Those whose children were scheduled to undergo a complex surgery and those who lived far from the hospital searched for information online more frequently. Most parents searched for information on the care provider’ website and, through the present survey, provided insights that can guide an improvement of information provided online on the hospital’s website. Two thirds of parents reported that online information increased their understanding regarding the surgery, even if this proportion was lower compared to other studies, thus prompting an improvement of the quality of information provided online, and suggesting a specific guidance by physicians on online information sources. One third of parents reported an improvement of the management of their child’s condition, showing a need of online contents providing practical guidance to parents.

## Data Availability

The datasets generated during and/or analysed during the current study are not publicly available due to hospital research policy and lack of consent from those interviewed. Aggregate analyses different from those presented in the article are however available on reasonable request to the corresponding author.

## Abbreviations

ICT: Information and communication technologies
AHQR: Agency for Healthcare and quality
OPBG: Bambino Gesù Children’s Hospital
ORS: Open Route Services

## References

[1] Eurostat. Digital economy and society statistics - households and individuals. https://ec.europa.eu/eurostat/statistics-explained/pdfscache/33472.pdf. Accessed 09 Apr 2020.

[2] ISTAT Cittadini, imprese e ICT 2017. https://www.istat.it/it/archivio/207825. Accessed 09 Apr 2020.

[3] NUA Internet surveys | Pambazuka News. https://www.pambazuka.org/security-icts/nua-internet-surveys-0. Accessed 09 Apr 2020.

[4] Rice RE (2006). Influences, usage, and outcomes of internet health information searching: multivariate results from the pew surveys. Int J Med Inf.

[5] Khoo K, Bolt P, Babl FE, Jury S, Goldman RD (2008). Health information seeking by parents in the internet age. J Paediatr Child Health.

[6] Goldman RD, Macpherson A (2006). Internet health information use and e-mail access by parents attending a paediatric emergency department. Emerg Med J.

[7] Wainstein BK, Sterling-Levis K, Baker SA, Taitz J, Brydon M (2006). Use of the internet by parents of paediatric patients. J Paediatr Child Health.

[8] Bouche G, Migeot V (2008). Parental use of the internet to seek health information and primary care utilisation for their child: a cross-sectional study. BMC Public Health.

[9] Jaks R, Baumann I, Juvalta S, Dratva J (2019). Parental digital health information seeking behavior in Switzerland: a cross-sectional study. BMC Public Health.

[10] Hand F, Mc Dowell DT, Glynn RW (2013). Patterns of internet use by parents of children attending a pediatric surgical service. Pediatr Surg Int.

[11] Bianco A, Zucco R, Nobile CG, Pileggi C, Pavia M (2013). Parents seeking health-related information on the internet: cross-sectional study. J Med Internet Res.

[12] Bernhardt JM, Felter EM (2004). Online pediatric information seeking among mothers of young children: results from a qualitative study using focus groups. J Med Internet Res.

[13] Nordfeldt S, Johansson C, Carlsson E, Hammersjö JA (2005). Use of the internet to search for information in type 1 diabetes children and adolescents: a cross-sectional study. Technol Health Care.

[14] Sim NZ, Kitteringham L, Spitz L, Pierro A, Kiely E, Drake D, Curry J (2007). Information on the world wide web—how useful is it for parents?. J Pediatr Surg.

[15] Wong MKY, Sivasegaran D, Choo CSC, Nah SA (2018). Parental internet use and health information seeking behavior comparing elective and emergency pediatric surgical situations. Eur J Pediatr Surg.

[16] Semere W, Karamanoukian HL, Levitt M, Edwards T, Murero M, D'Ancona G, Donias HW, Glick PL (2003). A pediatric surgery study: parent usage of the internet for medical information. J Pediatr Surg.

[17] Walsh AM, Hamilton K, White KM, Hyde MK (2015). Use of online health information to manage children's health care: a prospective study investigating parental decisions. BMC Health Serv Res.

[18] Malone M, While A, Roberts J (2014). Parental health information seeking and re-exploration of the 'digital divide'. Prim Health Care Res Dev.

[19] Sebelefsky C, Voitl J, Karner D, Klein F, Voitl P, Böck A (2016). Internet use of parents before attending a general pediatric outpatient clinic: does it change their information level and assessment of acute diseases?. BMC Pediatr.

[20] Sillence E, Briggs P (2007). Please advise: using the internet for health and financial advice. Comput Human Behav.

[21] Scullard P, Peacock C, Davies P (2010). Googling children’s health: reliability of medical advice on the internet. Arch Dis Childhood.

[22] Pandolfini C, Impicciatore P, Bonati M (2000). Parents on the web: risks for quality management of cough in children. Pediatrics..

[23] Fast AM, Deibert CM, Hruby GW, Glassberg KI (2013). Evaluating the quality of internet health resources in pediatric urology. J Pediatr Urol.

[24] Pehora C, Gajaria N, Stoute M, Fracassa S, Serebale-O'Sullivan R, Matava CT (2015). Are parents getting it right? A survey of parents’ internet use for children’s health care information. Interact J Med Res.

[25] Wong SSM, Wong KPL, Angus MIL, Chen Y, Choo CSC, Nah SA (2020). A pilot study comparing parent and adolescent online health information seeking behaviours in elective pediatric surgical situations. Pediatr Surg Int.

[26] Glynn RW, O’Duffy F, O’Dwyer TP, Colreavy MP, Rowley HM (2013). Patterns of internet and smartphone use by parents of children attending a pediatric otolaryngology service. Int J Pediatr Otorhinolaryngol.

[27] Ciofi Degli Atti ML, Serino L, Piga S, Tozzi AE, Raponi M (2017). Incidence of surgical site infections in children: active surveillance in an Italian academic children’s hospital. Ann Ig.

[28] Wald JS, Haque SN, Rizk S, Webb JR, Brown S, Ebron S (2018). Enhancing health IT functionality for children: the 2015 children’s EHR format. Pediatrics..

[29] Perin A, Galbiati TF, Ayadi R, Gambatesa E, Orena EF, Riker NI, et al. Informed consent through 3D virtual reality: a randomized clinical trial. Acta Neurochir (Wien). 2020. 10.1007/s00701-020-04303-y. 10.1007/s00701-020-04303-y 32242272

[30] Park E, Kwon M (2018). Health-related internet use by children and adolescents: systematic review. J Med Internet Res.

